# Health Insurance Economics

**Published:** 1917-06

**Authors:** 


### Health Insurance Economics.

Let us keep clearly before us that the main issue in Health Insurance and all similar plans affecting the profession is the economic one. It makes little difference to the average practitioner whether he gets his work and pay by competition after the analogy of the small business man or whether he serves as an official of some form of government, on a salary or fee basis. There is no immediate danger of interfering with the maintenance of more or less specialized practice on the competitive system by those who can get or are willing to take the chance of getting more than the average practitioner. There is little danger that the average physician will work harder or under greater compulsion to care for undesirable and non-remunerative cases, under government regulation than under his own sense of duty. The vital issue for the profession is its own compensation.

There is one fundamental basis on which any form of labor will receive its proper reward, barring a state of autocracy bordering on slavery, which need not be feared in this country. This is to be able to decline any offer of unjustly low pay or unjustly excessive hours and conditions of labor. This power, in turn, depends upon a genuine fulfillment of the law of supply and demand. Arbitrary and temporary substitutes may be employed but, in the long run, the law of supply and demand for anything, is inexorable. Just so long as there are more physicians than are needed, so long will the profession be endangered by threats of socialization.

